# Intra-therapeutic dosimetry of [^177^Lu]Lu-PSMA-617 in low-volume hormone-sensitive metastatic prostate cancer patients and correlation with treatment outcome

**DOI:** 10.1007/s00259-021-05471-4

**Published:** 2021-07-04

**Authors:** Steffie M. B. Peters, Bastiaan M. Privé, Maarten de Bakker, Frank de Lange, Walter Jentzen, Annemarie Eek, Constantijn H. J. Muselaers, Niven Mehra, J. Alfred Witjes, Martin Gotthardt, James Nagarajah, Mark W. Konijnenberg

**Affiliations:** 1 Department of Medical Imaging, Radboud University Medical Center, P.O. Box 9101, 6500 HB Nijmegen, The Netherlands; 2 Department of Nuclear Medicine, University of Duisburg-Essen, Essen, Germany; 3 Department of Urology, Radboud University Medical Center, Nijmegen, The Netherlands; 4 Department of Medical Oncology, Radboud University Medical Center, Nijmegen, The Netherlands; 5 Department of Radiology and Nuclear Medicine, Erasmus Medical Center, Rotterdam, The Netherlands

**Keywords:** [^177^Lu]Lu-PSMA, Dosimetry, Radionuclide therapy, Prostate cancer, mHSPC

## Abstract

**Introduction:**

While [^177^Lu]Lu-PSMA radioligand therapy is currently only applied in end-stage metastatic castrate-resistant prostate cancer (mCRPC) patients, also low-volume hormone-sensitive metastatic prostate cancer (mHSPC) patients can benefit from it. However, there are toxicity concerns related to the sink effect in low-volume disease. This prospective study aims to determine the kinetics of [^177^Lu]Lu-PSMA in mHSPC patients, analyzing the doses to organs at risk (salivary glands, kidneys, liver, and bone marrow) and tumor lesions < 1 cm diameter.

**Methods:**

Ten mHSPC patients underwent two cycles of [^177^Lu]Lu-PSMA therapy. Three-bed position SPECT/CT was performed at 5 time points after each therapy. Organ dosimetry and lesion dosimetry were performed using commercial software and a manual approach, respectively. Correlation between absorbed index lesion dose and treatment response (PSA drop of > 50% at the end of the study) was calculated and given as Spearman’s r and p-values.

**Results:**

Kinetics of [^177^Lu]Lu-PSMA in mHSPC patients are comparable to those in mCRPC patients. Lesion absorbed dose was high (3.25 ± 3.19 Gy/GBq) compared to organ absorbed dose (salivary glands: 0.39 ± 0.17 Gy/GBq, kidneys: 0.49 ± 0.11 Gy/GBq, liver: 0.09 ± 0.01 Gy/GBq, bone marrow: 0.017 ± 0.008 Gy/GBq). A statistically significant correlation was found between treatment response and absorbed index lesion dose (*p* = 0.047).

**Conclusions:**

We successfully performed small lesion dosimetry and showed that the tumor sink effect in mHSPC patients is of less concern than was expected. Tumor-to-organ ratio of absorbed dose was high and tumor uptake correlates with PSA response. Additional treatment cycles are legitimate in terms of organ toxicity and could lead to better tumor response.

**Supplementary Information:**

The online version contains supplementary material available at 10.1007/s00259-021-05471-4.

## Introduction

Prostate cancer is the second most common cancer worldwide, with over 1.3 million patients diagnosed every year [1, 2]. While survival is good in patients diagnosed in an early stage eligible for curative surgery or external beam radiotherapy, the prognosis of patients in advanced disease is poor [3, 4]. Prostate-specific membrane antigen (PSMA) is a transmembrane protein highly overexpressed in about 90% of prostate cancers, and is positively correlated with level of expression and aggressiveness of the disease [5–8]. Therefore, PSMA is considered an ideal target for molecular imaging and therapy of prostate cancer [9–13].

In recent years, [^177^Lu]Lu-PSMA-617 radioligand ([^177^Lu]Lu-PSMA) treatment is increasingly applied to end-stage metastatic castrate-resistant prostate cancer (mCRPC) patients with remarkable responses coupled with a favorable toxicity profile [14–22]. While various radionuclides are available for therapeutic application, ^177^Lu is particularly useful as the beta emission delivers tumoricidal absorbed doses in a range of 1–2 mm, while its gamma component allows for imaging and quantification with SPECT/CT, providing input for absorbed dose calculations. These dosimetry studies performed in end-stage disease found high absorbed doses of [^177^Lu]Lu-PSMA to tumors and marked the salivary glands, lacrimal glands, kidneys, and bone marrow as organs at risk [16, 20, 23–26].

Currently, [^177^Lu]Lu-PSMA is only applied in high-volume mCRPC, but it is anticipated that patients in earlier stages could also benefit from this therapy. To date, only one prospective clinical trial was carried out applying [^177^Lu]Lu-PSMA in low-volume hormone-sensitive metastatic prostate cancer (mHSPC) patients and revealed it to be a feasible and safe treatment modality [27]. Yet, in contrast to mCRPC patients, mHSPC patients have a longer survival with several good treatment options available. This warrants more careful assessment of treatment efficacy and toxicity, which could be assured by dosimetry. Moreover, in these low-volume disease patients, there are concerns regarding the tumor sink effect, hypothesizing that low tumor volume could lead to unfavorable radioligand distribution to the organs at risk [28, 29]. To date, no elaborate dosimetry study was performed in this early-stage patient cohort, so the pharmacokinetics of [^177^Lu]Lu-PSMA in mHSPC patients are still unknown. Also, it is still unclear what the efficacy is of [^177^Lu]Lu-PSMA in small tumor metastases (< 1 cm) since it is challenging to perform dosimetry on such small lesions and the currently available software methods are mainly appropriate to reliably assess dose to larger lesions. Information from dosimetry studies in end-stage patients has been less elaborate, either using fewer time points, missing 3D data, or focusing on just one treatment cycle. Hence, this is the first study, embedded in the abovementioned prospective study [27], presenting all dosimetry results including the smallest lesions detected by PET and organs at risk (salivary glands, kidney, liver, and bone marrow), using a state-of-the-art dosimetry protocol. Moreover, the tumor absorbed doses were compared between treatment cycles and correlated to the observed clinical responses.

## Methods

### Study design and patient population

Ten patients with low-volume mHSPC were enrolled between September 2018 and October 2019. Inclusion criteria were a prostate-specific antigen (PSA) doubling time ≤ 6 months and ≤ 10 metastatic lesions on [^68^Ga]Ga-PSMA-11 PET/CT ([^68^Ga]Ga-PSMA-PET/CT), with at least one lesion ≥ 1.0 cm in diameter to enable more precise dosimetry. Based on the [^68^Ga]Ga-PSMA-PET/CT signal, an index lesion was defined for each patient (taking into account SUV_max_ value as well as suitable lesion size for dosimetry). Patients were not allowed to have received prior hormonal therapy (except for temporary neo-adjuvant androgen deprivation therapy combined with external beam radiotherapy for localized prostate cancer) or chemotherapy. Patients eligible for local treatments for oligometastatic diseases (e.g., salvage radiotherapy or surgery) were excluded from the study. This study was approved by the Medical Review Ethics Committee Region Arnhem-Nijmegen and was registered on ClinicalTrials.gov (NCT03828838). All subjects provided written informed consent before study entry.

The study flowchart can be found in Online Resource Figure S1. All patients underwent two cycles of [^177^Lu]Lu-PSMA therapy. The preparation of [^177^Lu]Lu-PSMA-617 has been previously described [27]. The activity was administered by slow intravenous injection over 2–5 min. Patients were advised to have adequate oral fluid intake next to a NaCl 0.9% infusion of two l per 24 h. No specific actions were taken to prevent xerostomia. In the first cycle, a therapeutic activity of 3 GBq of [^177^Lu]Lu-PSMA was administered. For the second cycle, the administered activity was adjusted between 3 and 6 GBq depending on the dosimetry results derived from the first cycle for organs at risk and index lesions. Toxicity (hematology, renal/liver functions, xerostomia) and PSA level were monitored weekly until 12 weeks after the second cycle and followed up until the end of the study at week 24 after the second cycle (EOS). Clinical results have been described by Privé et al. [27].

### Image acquisition

[^68^Ga]Ga-PSMA-PET/CT imaging was performed on a Biograph mCT system (Siemens Healthineers, Erlangen, Germany) 1 week prior to each administration of [^177^Lu]Lu-PSMA to evaluate PSMA-positive tumor lesions, following local clinical acquisition protocols [30]. Standardized uptake values (SUV) were determined for salivary gland (SUV_mean_, spherical volumes of interest (VOIs) of 20 mm in diameter) and lesions (SUV_max_). For dosimetry, all patients received SPECT/CT imaging at 1, 24, 48, 72, and 168 h after each therapy on either a Symbia T16 or Symbia Intevo Bold system (Siemens Healthineers, Erlangen, Germany). Both systems were cross-calibrated for ^177^Lu with the in-house dose calibrator, which undergoes regular quality control according to national guidelines [31]. Three-bed position SPECT/CT scans were acquired including the pelvis, abdomen, and head/neck region. The acquisition and reconstruction protocol was followed as described by Peters et al. [32] and was in accordance with MIRD pamphlet no. 26 [33]. These protocols take into account scatter, attenuation, and dead-time corrections.

### Organ dosimetry

Volumetric organ-based dosimetry was performed according to the scheme defined by the Committee on Medical Internal Radiation Dose (MIRD) [34] using Hermes HybridViever/Dosimetry (Hermes Medical Solutions, Stockholm, Sweden) for salivary glands, kidneys, and liver. Absorbed dose was calculated using the MIRD equation:

1 $$D(mGy)=\tilde{A} \left( MBq\cdot h\right)\cdot S\left( mGy/ MBq\cdot h\right)$$

where *D* is the absorbed dose, *Ã* is the cumulated activity, and *S* is the common S-value for physical effects. All scans were co-registered and VOIs were drawn based on CT contours of organs. For the salivary glands, a VOI including the organ plus a ~ 1 cm margin was selected to account for partial volume effects. Fitting of data for determination of the time-activity curve and cumulated activity in Hermes means assuming instantaneous uptake between t = 0 and the first imaging time point, trapezoidal integration between the first imaging time point and the first fit time point selected by the user, mono- or bi-exponential fitting between first fit time point and last imaging time point, and extrapolation of the curve from the last imaging time point to infinity. Table 1 gives an overview of the specific fit conditions for each organ and its respective standard mass used.

**Table 1:** Fit conditions per organ structure. For comparison, the tumor fit conditions and S-values are included

Structure	Type of fit	First fit point (p.i.)	Organ weight (g)	S-value (mGy/MBq·s)
Salivary glands	Bi-exponential	24 h	85	2.78E-4
Kidneys	Mono-exponential	1 h	310	7.76E-5
Liver	Mono-exponential	1 h	1800	1.37E-5
Bone marrow	Three-exponential	5 min	1170	1.15E-5
Tumor	Mono-exponential	24 h	0.1	2.21E-1
			0.5	4.57E-2
			1.0	2.31E-2

*p.i.* post injection

Organ absorbed dose *D* was determined in Olinda 2.1 (Hermes Medical Solutions, Stockholm, Sweden) using organ weights based on the ICRP Publication 89 adult male human model [35] (without mass scaling) and corresponding S-values.

### Bone marrow dosimetry

Bone marrow dosimetry was performed according to the EANM Dosimetry Committee guidelines for bone marrow and whole-body dosimetry [36]. No active uptake in bone or bone marrow was assumed since this was also not seen in mCRPC patients [14, 20, 23–25]; therefore, the blood sampling method was used. After each therapy, blood draws were collected at 5, 30, 60, 120, and 180 min and 1, 2, 3, and 7 days post injection. Blood samples were measured in a scintillation counter (248 WIZARD^2^, PerkinElmer, Groningen, The Netherlands) that was calibrated for ^177^Lu to translate from counts per minute (CPM) to megabecquerels (MBq) per volume unit (ml). Time-activity curves were fitted to a three-exponential decay using GraphPad Prism 5.03 (GraphPad Software Inc., CA, USA).

### Lesion dosimetry

This study focused on small lesion dosimetry (mostly < 1 ml volume on [^68^Ga]Ga-PSMA-PET/CT). Dosimetry of such small structures is challenging and not straightforward due to limitations in scanner resolution and sensitivity, introducing significant partial volume effects and high uncertainty in absorbed dose calculations. Therefore, the dosimetry protocol was optimized to minimize the uncertainty as much as possible. First, a slightly oversized VOI (20–30% larger than the visible structure) was drawn for the lesions on SPECT/CT in Hermes dosimetry to account for partial volume effects of small structures. Background correction was applied by drawing a VOI close to the lesion VOI and subtract background counts from lesion counts, considering the volumes of lesion and background VOI [37]. To determine the cumulated activity, a combined approach was used: a trapezoid method between time points 0 and 24 h, and a mono-exponential model without residual activity thereafter (using GraphPad Prism 5.03). In case the correlation coefficient (R^2^) of the fit was below 0.7, the goodness of fit was considered too low and the trapezoid method was used between all time points. In that case, the tail was determined by extrapolating the effective half-life between the last two acquired data points.

Lesion volumes were determined on the [^68^Ga]Ga-PSMA-PET/CT acquired 1 week prior to therapy. An anatomical slice by slice approach on the (low-dose) CT image was used if possible. Otherwise, an iterative thresholding method using the PET signal was used as suggested by Jentzen [38]. Lesion dose was determined using the IDAC-Dose 2.1 sphere model S-values [39] for a corresponding sphere volume of water.

### Statistical analysis

All dosimetry data are indicated per patient including the uncertainty in the absorbed dose values following the EANM uncertainty guideline by Gear et al. [40] (for more details, see Online Resource Materials S1). For organs, uncertainty in absorbed dose is mainly the result of the uncertainty in the time-activity curve; therefore, it was assumed to be directly proportional to the cumulated activity and other uncertainties were ignored. For lesions, also the uncertainty in volume determination was taken into account. For combined statistics of all patients, data are given in median, range, mean, and standard deviation. Correlations between salivary gland/lesion SUV on [^68^Ga]Ga-PSMA-PET/CT and absorbed dose after therapy were calculated and given as Spearman’s r and p-values, as well as correlation between lesion volume change and absorbed dose. Difference between absorbed dose after the first and second cycle was assessed using a Wilcoxon matched-pairs signed ranks test. A Wilcoxon-Mann–Whitney test was used to test for difference in absorbed dose to soft tissue and bone lesions, as well as for difference in absorbed dose to tumor in patients achieving a PSA response below or over 50%. A p-value < 0.05 is considered statistically significant. All analyses were performed in GraphPad Prism 5.03.

## Results

Patient characteristics, administered activities (GBq), and PSA response at the end of the study can be found in Table 2. The administered activity of the second cycle was set to 6 GBq for all patients to maximize the absorbed dose to the tumors, as none of the organs received toxic doses in the first cycle. The median administered activity for both cycles was 9.0 GBq (range 8.0–9.2 GBq). None of the patients showed kidney, liver, or bone marrow toxicity in their blood measurements after each therapy cycle (Online Resource Figure S2). Two out of ten patients reported mild xerostomia after two treatment cycles, which had disappeared at the end of the study [27].

**Table 2:** Patient characteristics and administered activities

Patient #	Age (years)	Weight (kg)	PSA response (%)	Activity cycle 1 (GBq)	Activity cycle 2 (GBq)
1	61	89	> 50	3.0	6.0
2	62	91	< 50	3.1	6.1
3	77	77	> 50	3.1	6.0
4	66	96	< 50	3.1	6.0
5	68	78	> 50	3.0	6.0
6	65	86	< 50	3.0	6.0
7	71	75	< 50	3.1	4.9
8	71	59	> 50	3.1	6.0
9	69	90	> 50	3.0	6.0
10	62	85	< 50	3.0	6.0

### Organ dosimetry

The median effective half-life (T_1/2,eff_) of [^177^Lu]Lu-PSMA was 32.5 h for salivary glands (range: 23.9–42.2 h), 28.4 h for kidneys (range: 15.0–46.5 h), and 19.0 h for liver (range: 12.4–23.2 h). The kinetics per organ for both cycles can be found in Online Resource Figures S3, S4, and S5. The time integrated activity (MBq·h/MBq) was not significantly different between the first and second cycles. Blood clearance kinetics showed a three-phase decay with a fast component with a median half-life of 11.1 min, an intermediate component with a half-life of 2.7 h, and a slow component of 12.5 h. Calculated absorbed doses for each organ and patient can be found in Online Resource Table S1 and are summarized in Table 3. The standard deviation in absorbed dose between patients (0.17, 0.11, 0.01, and 0.008 Gy/GBq for salivary glands, kidney, liver, and bone marrow, respectively) was larger than the intra-patient uncertainty (0.06, 0.04, and 0.04 Gy/GBq). Organs with the highest absorbed dose are salivary glands and kidneys.

**Table 3:** Absorbed dose in organs at risk (Gy/GBq)

	Salivary glands	Kidneys	Liver	Bone marrow
Mean + SD	0.39 ± 0.17	0.49 ± 0.11	0.09 ± 0.01	0.017 ± 0.008
Median	0.38	0.49	0.09	0.018
Range	0.14–0.66	0.34–0.66	0.07–0.12	0.013–0.023

*SD* standard deviation

**Fig. 1:**
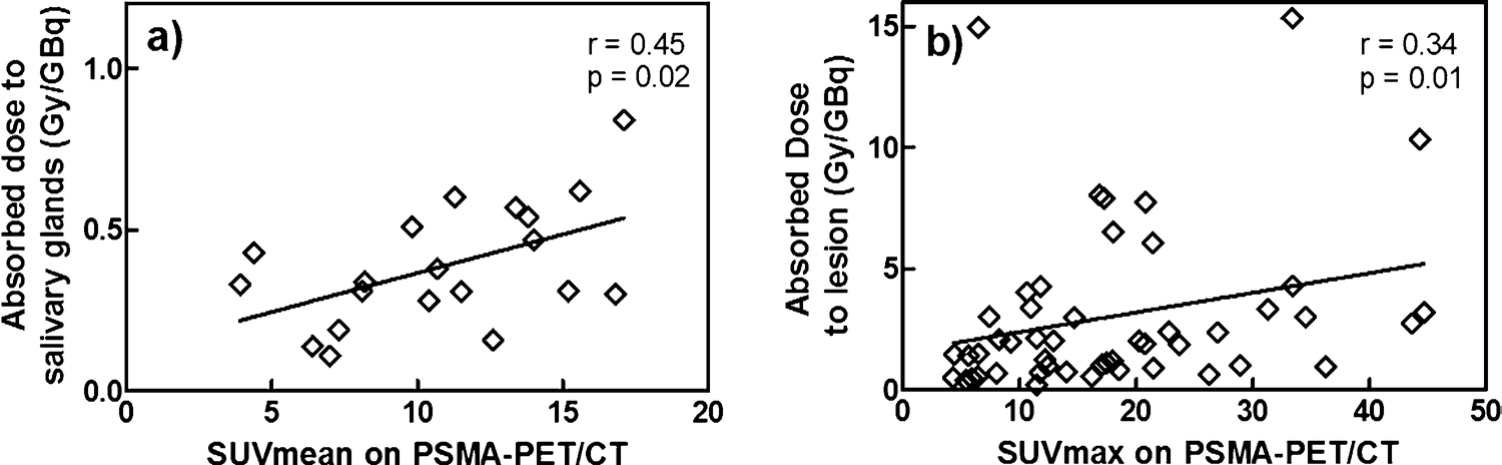
Correlation between SUV_mean/max_ on [^68^Ga]Ga-PSMA-PET/CT and absorbed dose in salivary glands (**a**) and lesions (**b**). Data of treatment cycles 1 and 2 are combined

Higher uptake in the salivary glands on pre-therapeutic [^68^Ga]Ga-PSMA-PET (SUV_mean_) correlated with higher absorbed dose per administered activity (r = 0.45, p = 0.02; Fig. 1a).

### Lesion dosimetry

**Fig. 2:**
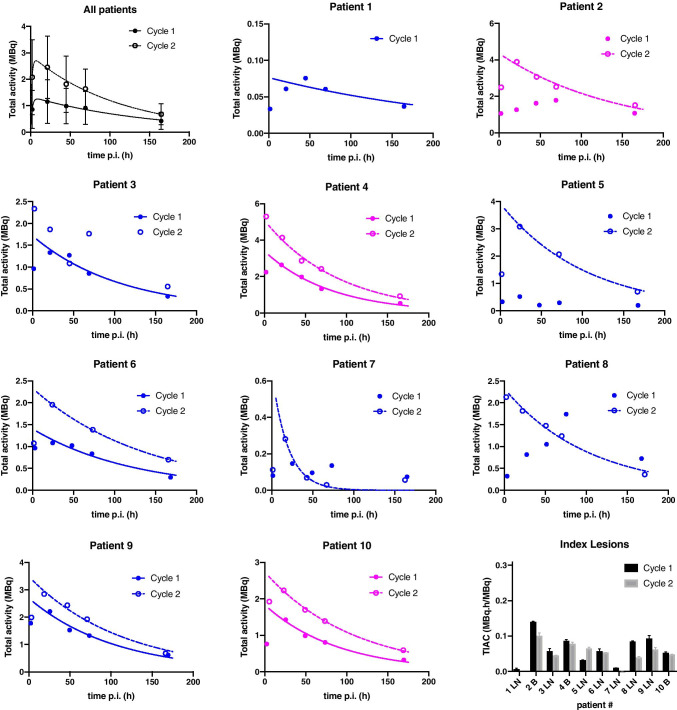
The [^177^Lu]Lu-PSMA kinetics per cycle for index lesions in each patient. Blue lines represent lymph node lesions; purple lines represent bone lesions. Mono-exponential fits with R^2^ < 0.7 were excluded, and in these cases a trapezoidal method was used to determine the TIAC. In these cases, the fits are not shown in this figure (*n* = 5)

**Fig. 3:**
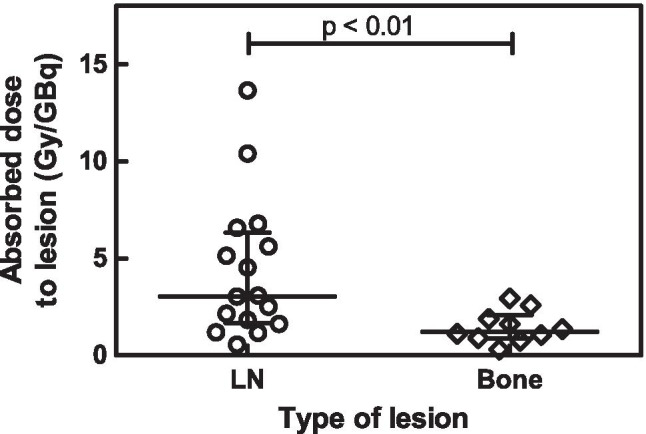
Absorbed dose in lymph node (LN) lesions was significantly higher than in bone lesions

Patients had between one and seven metastases that were assessed by dosimetry. A total of 16 lymph node lesions and 10 bone lesions were analyzed. The median clearance half-life was 62 h (range: 13–417 h, Fig. 2). The median volume was 0.68 ml (range: 0.05–42.5 ml) with an average uncertainty of 10%. The absorbed dose per lesion was 2.0 Gy/GBq for all lesions together (median, range: 0.3–13.7) (Table 4 and Online Resource Table S2). The lesion absorbed dose per activity after the second cycle was not significantly different from the lesion absorbed dose after the first cycle (p = 0.25). Absorbed dose in lymph node lesions (median: 3.1 Gy/GBq, range: 0.6–13.7) was significantly higher than in bone lesions (median: 1.1 Gy/GBq, range: 0.3–3.1, *p* < 0.01) (Fig. 3), while lesion uptake on pre-therapeutic [^68^Ga]Ga-PSMA-PET (SUV_max_) was not significantly different for lymph node and bone lesions. However, SUV_max_ correlated with absorbed dose in the corresponding lesion (r = 0.34, p = 0.01; Fig. 1b). The higher absorbed dose in lymph node lesions corresponded with the observed volume change after the first cycle of therapy: most lymph node lesions decreased in size (35% mean volume decrease) whereas most bone lesions increased in size (36% mean volume increase). However, correlation between absorbed dose and lesion volume change was not significant (r =  − 0.23, p = 0.23). The average uncertainty in cumulated activity was 22%. For the absorbed dose, dependent on lesion volume and cumulated activity, the average uncertainty was 25%.

**Table 4:** Absorbed dose in lesions ((Gy/GBq) or (Gy))

Type of lesion	Number of lesions	Parameter	Cycle 1 (Gy/GBq)	Cycle 2 (Gy/GBq)	Total (Gy)
All	26	Median	1.69	2.08	14.66
		Range	0.41–10.34	0.24–15.35	1.74–123.48
Lymph node	16	Median	1.84	6.08	25.31
		Range	0.52–10.34	0.47–15.35	1.74–123.48
Bone	10	Median	1.48	1.11	11.24
		Range	0.41–3.67	0.24–2.75	2.75–28.23

**Fig. 4:**
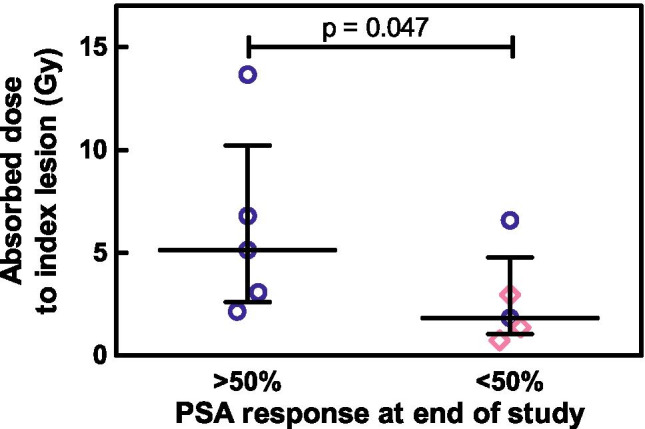
Absorbed dose to index lesion for patients showing a PSA response of > 50% at the end of the study was significantly higher than for patients showing a response of < 50%. Blue circles indicate lymph node lesions; purple diamonds indicate bone lesions

Treatment response (PSA drop of > 50% vs. < 50% at the end of the study) correlated with absorbed index lesion dose (p = 0.047, Fig. 4).

## Discussion

Absorbed dose of [^177^Lu]Lu-PSMA in organs was comparable to what was reported for high-volume mCRPC patients [16, 20, 23–26], indicating that organ kinetics for [^177^Lu]Lu-PSMA are more or less equal in both low-volume and high-volume metastatic patients. This confirms the physiologically based pharmacokinetic (PKPB) model finding by Begum et al. [41], indicating minimal influence of total lesion volume on the absorbed dose to kidneys and salivary glands by [^177^Lu]Lu-PSMA. However, Violet and colleagues found a correlation between tumor volume and absorbed dose in salivary glands in mCRPC patients [23]. This was also observed for [^68^Ga]Ga-PSMA-11, which showed a decrease in the order of 60% in SUV of salivary glands and kidneys for patients with high tumor load [29]. Thus, a more elaborative comparative study will be needed to elucidate the differential observations. Nonetheless, our data clearly showed that the sink effect in low-volume disease is of less concern than was expected and we were able to show a promising tumor-to-organ ratio of [^177^Lu]Lu-PSMA in these early-stage patients. We furthermore showed that the absorbed dose (Gy/GBq) in organs appeared to be similar or lower in the second cycle, which suggests that the tumor sink effect does not increase in later treatment cycles. This finding was supported by the result that the organ time integrated activity was not significantly different between cycles 1 and 2, indicating similar tracer biokinetics. The absence of organ toxicity [27] corresponded well with the absorbed dose found in all organs, which remained below any threshold dose for radiation-induced tissue effects [42–45]. Taking into account the range of absorbed dose (Gy/MBq) for each organ, our data suggest that a total activity up to at least 38 GBq [^177^Lu]Lu-PSMA is safe regarding the organs at risk (Online Resource Table S3). Moreover, these tolerance doses are mostly determined and used in external beam radiotherapy, whereas it is known that tissues can tolerate higher doses at the low dose rates associated with radionuclide therapy. This indicates that additional treatment cycles and/or higher injected activity per cycle are feasible to achieve higher tumor dose without risking negative effects to organs. However, no significant acute organ toxicity was found so correlations with tissue absorbed dose are not informative, as it falls within the constant (background) level of the sigmoid dose–response curve. Additionally, to date, no information is available on late occurring effects in for example kidneys, which is relevant in mHSPC patients because of their relatively long survival.

For bone marrow dosimetry, no active uptake in bone and bone marrow was assumed. Although some patients had bone metastases, these did not involve significant sections of the bone marrow. However, the blood sampling method might not be suitable if larger osseous areas are affected by tracer uptake, such as in high-volume (bone) disease.

In this study, we performed SPECT dosimetry of lesions with < 1 cm in diameter after [^177^Lu]Lu-PSMA therapy, which has not been described in literature to date. SPECT/CT dosimetry of smaller lesions is challenging because the assessment of tumor volume and cumulated activity is complicated. This introduces uncertainty to the absorbed dose, especially using protocolized software. Therefore, we optimized the methodology to determine absorbed dose in small lesions. Cumulated activity was not determined in commercially available software but using an in-house developed method which enabled the application of background correction and more freedom in the fitting method, leading to a more precise estimation. Lesion volume was manually assessed slice by slice on [^68^Ga]Ga-PSMA-PET/CT, leading to a more reliable volume estimation than based on SPECT signal. We also compared our final tumor volumes to volumes determined by measuring the lesion diameter on CT and calculating the volume assuming a spherical or cubical model for lymph node or bone metastases, respectively. This resulted in a similar mean lesion volume (3.45 ml vs. 3.72 ml for our method), but the uncertainty increased from 10 to 30%. Lack of background correction and less precise methodology for volume determination lead to an uncertainty in absorbed dose of around 43%, as compared to 25% in the present study. Using commercially available MIRD software may therefore serve to roughly estimate the absorbed doses in small lesions, as was reported by Privé et al. [27], but one needs to be aware of the significant increase in uncertainty when using these methods. This might be especially relevant when the absorbed tumor dose is used for clinical decision-making in terms of further treatment planning. Also, more reliable dose estimations could potentially help to correlate absorbed lesion dose to clinical outcomes.

While the five different time point whole-body SPECT/CT imaging enabled accurate dosimetry, the clinical translation of the present protocol is unlikely as it is time-consuming and requires considerable effort and resources from patients and the clinics. Therefore, there is a need to perform dosimetry using a simplified yet reliable protocol.

One such option for simplification was provided by assessing index lesions. In high-volume disease with numerous metastases, the absorbed dose of a single index lesion might not reflect the response accurately due to tumor heterogenicity, as was indeed found for mCRPC patients. In these patients, a significant correlation between total lesion volume absorbed dose and PSA response was observed, but not when considering index lesions only [23]. However, in the present study with low-volume mHSPC patients, less cancer heterogenicity between metastases exists [46–48]. This was confirmed by a significant correlation between index lesion absorbed dose and PSA response in our study. Thus, single index lesion dosimetry could serve as a good indicator of expected treatment outcome in low-volume disease and a one-bed position SPECT/CT (per time point) might suffice for future studies and clinical translation.

Additionally, we compared absorbed dose is organs and lesions between cycles and found that it might be feasible to limit an elaborate dose estimation to the first cycle. Additional cycles could then be evaluated by acquiring a SPECT/CT at one time point and use kinetical information from the first cycle to estimate the absorbed dose using a simplified approach according to Hänscheid and colleagues [49] (Additional Resource Materials S2). For example, for the salivary glands, the 24 or 48 h time point could be sufficient to get a reliable dose estimation for the second cycle. For the lesions however, the correlation was less evident and additional time points might be necessary. Further studies to develop such a protocol are warranted.

We observed that soft tissue lesions in this patient cohort responded significantly better to radioligand therapy than bone lesions, which was also reflected in the corresponding volume and PSA change. This is in line with what was found in mCRPC patients [50]. In early-stage prostate cancer patients, treatment with ^177^Lu-PSMA is expected to be especially beneficial in patients that predominantly have soft tissue lesions.

Furthermore, it was confirmed that PSMA-PET SUV can be accurately used for patient selection, since both salivary glands and lesion SUV correlated with the absorbed dose, again similar to the findings in mCRPC patients [23]. Moreover, we showed that even single lesion SUV_max_ (instead of total lesion volume) correlated with the absorbed dose in the corresponding lesion. This information could be useful for [^177^Lu]Lu-PSMA patient selection.

While there is uncertainty in absorbed dose for organs at risk and lesions, the standard deviation between patients was larger than the intra-patient uncertainty. Especially in the lesions, we found individual differences in [^177^Lu]Lu-PSMA kinetics (Fig. 2). This suggests that our patient-specific dosimetry calculations are reliable enough to use for a personalized approach in the dosing scheme in this early-stage patient cohort, just like is recommended for patients receiving ^177^Lu-octreotate peptide receptor therapy for neuroendocrine tumors [51]. Of course, our results are based on a small number of patients and lesions, so further studies on larger patient numbers are warranted to confirm these findings. Based on the current results, our perspective is standardization of administered activity in the first cycle whereas the following cycles are based on the dosimetry results of the first cycle. This way, therapeutic efficacy can be verified while preventing healthy organ toxicity, and individuals showing low tumoricidal doses can be recommended for an alternative therapeutic strategy.

## Conclusion

In this prospective dosimetry study in low-volume mHSPC patients, we showed that the kinetics of [^177^Lu]Lu-PSMA-617 are comparable to those in high-volume mCRPC patients. [^177^Lu]Lu-PSMA showed promising tumor-to-organ ratio in these early-stage patients. None of the organs at risk reached threshold radiation doses whereas tumor absorbed dose was high, including the smallest metastases detected by PET imaging. Additional treatment cycles are possible in terms of organ toxicity and could lead to even better lesion response. Dosimetry can help to individualize the treatment plan in these early-stage patients. Studies that optimize the dosimetry protocol for clinical translation are warranted.

## Supplementary Information

Below is the link to the electronic supplementary material.

Supplementary file1 (DOCX 1215 KB)

## Data Availability

The datasets generated during and/or analyzed during the current study are available from the corresponding author on reasonable request.

## Abbreviations

CPM: Counts per minute
CT: Computed tomography
EANM: European Association of Nuclear Medicine
EOS: End of cycle
LN: Lymph node
mCRPC: Metastasized castrate-resistant prostate cancer
MIRD: Medical Internal Radiation Dose
mHSPC: Metastasized hormone-sensitive prostate cancer
NaCl: Natriumchloride
PET: Positron emission tomography
p.i.: Post injection
PKPB: Physiologically based pharmacokinetic model
PSA: Prostate-specific antigen
PSMA: Prostate-specific membrane antigen
SD: Standard deviation
SPECT: Single-photon emission computed tomography
SUV: Standardized uptake values
TIA: Time integrated activity
TIAC: Time integrated activity curve
VOI: Volume of interest

## References

[1] Schröder FH, Hugosson J, Roobol MJ, Tammela TL, Ciatto S, Nelen V (2012). Prostate-cancer mortality at 11 years of follow-up. N Engl J Med.

[2] Bray F, Ferlay J, Soerjomataram I, Siegel RL, Torre LA, Jemal A (2018). Global cancer statistics 2018: GLOBOCAN estimates of incidence and mortality worldwide for 36 cancers in 185 countries. CA Cancer J Clin.

[3] Cornford P, Bellmunt J, Bolla M, Briers E, De Santis M, Gross T (2017). EAU-ESTRO-SIOG guidelines on prostate cancer. Part II: treatment of relapsing, metastatic, and castration-resistant prostate cancer. Eur Urol.

[4] James ND, Spears MR, Clarke NW, Dearnaley DP, De Bono JS, Gale J (2015). Survival with newly diagnosed metastatic prostate cancer in the “docetaxel era”: data from 917 patients in the control arm of the STAMPEDE trial (MRC PR08, CRUK/06/019). Eur Urol.

[5] Wright GL, Haley C, Beckett ML, Schellhammer PF (1995). Expression of prostate-specific membrane antigen in normal, benign, and malignant prostate tissues. Urol Oncol..

[6] Perner S, Hofer MD, Kim R, Shah RB, Li H, Möller P (2007). Prostate-specific membrane antigen expression as a predictor of prostate cancer progression. Hum Pathol.

[7] Silver DA, Pellicer I, Fair WR, Heston W, Cordon-Cardo C (1997). Prostate-specific membrane antigen expression in normal and malignant human tissues. Clin Cancer Res.

[8] Privé BM, Israël B, Schilham MG, Muselaers CH, Zámecnik P, Mulders PF, et al. Evaluating F-18-PSMA-1007-PET in primary prostate cancer and comparing it to multi-parametric MRI and histopathology. Prostate Cancer Prostatic Dis. 2020: 1–8. 10.1038/s41391-020-00292-2 32999466

[9] Afshar-Oromieh A, Avtzi E, Giesel FL, Holland-Letz T, Linhart HG, Eder M (2015). The diagnostic value of PET/CT imaging with the 68 Ga-labelled PSMA ligand HBED-CC in the diagnosis of recurrent prostate cancer. Eur J Nucl Med Mol Imaging.

[10] Maurer T, Gschwend JE, Rauscher I, Souvatzoglou M, Haller B, Weirich G (2016). Diagnostic efficacy of 68gallium-PSMA positron emission tomography compared to conventional imaging for lymph node staging of 130 consecutive patients with intermediate to high risk prostate cancer. J Urol.

[11] Giesel FL, Knorr K, Spohn F, Will L, Maurer T, Flechsig P (2019). Detection efficacy of 18F-PSMA-1007 PET/CT in 251 patients with biochemical recurrence of prostate cancer after radical prostatectomy. J Nucl Med.

[12] Kesch C, Kratochwil C, Mier W, Kopka K, Giesel FL (2017). 68Ga or 18F for prostate cancer imaging?. J Nucl Med.

[13] Pandit-Taskar N, O’Donoghue JA, Ruan S, Lyashchenko SK, Carrasquillo JA, Heller G (2016). First-in-human imaging with 89Zr-Df-IAB2M anti-PSMA minibody in patients with metastatic prostate cancer: pharmacokinetics, biodistribution, dosimetry, and lesion uptake. J Nucl Med.

[14] Zechmann CM, Afshar-Oromieh A, Armor T, Stubbs JB, Mier W, Hadaschik B (2014). Radiation dosimetry and first therapy results with a 124 I/131 I-labeled small molecule (MIP-1095) targeting PSMA for prostate cancer therapy. Eur J Nucl Med Mol Imaging.

[15] Ahmadzadehfar H, Eppard E, Kürpig S, Fimmers R, Yordanova A, Schlenkhoff CD (2016). Therapeutic response and side effects of repeated radioligand therapy with 177Lu-PSMA-DKFZ-617 of castrate-resistant metastatic prostate cancer. Oncotarget.

[16] Baum RP, Kulkarni HR, Schuchardt C, Singh A, Wirtz M, Wiessalla S, et al. Lutetium-177 PSMA radioligand therapy of metastatic castration-resistant prostate cancer: safety and efficacy. J Nucl Med. 2016;57(7):1006–13. 10.2967/jnumed.115.168443 26795286

[17] Heck MM, Tauber R, Schwaiger S, Retz M, D’Alessandria C, Maurer T (2019). Treatment outcome, toxicity, and predictive factors for radioligand therapy with 177Lu-PSMA-I&T in metastatic castration-resistant prostate cancer. Eur Urol.

[18] Tagawa ST, Milowsky MI, Morris M, Vallabhajosula S, Christos P, Akhtar NH (2013). Phase II study of lutetium-177–labeled anti-prostate-specific membrane antigen monoclonal antibody J591 for metastatic castration-resistant prostate cancer. Clin Cancer Res.

[19] Rahbar K, Ahmadzadehfar H, Kratochwil C, Haberkorn U, Schäfers M, Essler M (2017). German multicenter study investigating 177Lu-PSMA-617 radioligand therapy in advanced prostate cancer patients. J Nucl Med.

[20] Kratochwil C, Giesel FL, Stefanova M, Benesova M, Bronzel M, Afshar-Oromieh A (2016). PSMA-targeted radionuclide therapy of metastatic castration-resistant prostate cancer with Lu-177 labeled PSMA-617. J Nucl Med.

[21] Hofman MS, Emmett L, Sandhu SK, Iravani A, Joshua AM, Goh JC (2020). TheraP: a randomised phase II trial of 177Lu-PSMA-617 (LuPSMA) theranostic versus cabazitaxel in metastatic castration resistant prostate cancer (mCRPC) progressing after docetaxel: initial results (ANZUP protocol 1603). J Clin Oncol.

[22] Hofman MS, Violet J, Hicks RJ, Ferdinandus J, Thang SP, Akhurst T (2018). [177Lu]-PSMA-617 radionuclide treatment in patients with metastatic castration-resistant prostate cancer (LuPSMA trial): a single-centre, single-arm, phase 2 study. Lancet Oncol.

[23] Violet JA, Jackson P, Ferdinandus J, Sandhu S, Akhurst T, Iravani A (2019). Dosimetry of Lu-177 PSMA-617 in metastatic castration-resistant prostate cancer: correlations between pre-therapeutic imaging and “whole body” tumor dosimetry with treatment outcomes. J Nucl Med..

[24] Okamoto S, Thieme A, Allmann J, D'Alessandria C, Maurer T, Retz M (2017). Radiation dosimetry for 177Lu-PSMA I&T in metastatic castration-resistant prostate cancer: absorbed dose in normal organs and tumor lesions. J Nucl Med.

[25] Delker A, Fendler WP, Kratochwil C, Brunegraf A, Gosewisch A, Gildehaus FJ (2016). Dosimetry for 177 Lu-DKFZ-PSMA-617: a new radiopharmaceutical for the treatment of metastatic prostate cancer. Eur J Nucl Med Mol Imaging.

[26] Kabasakal L, AbuQbeitah M, Aygün A, Yeyin N, Ocak M, Demirci E (2015). Pre-therapeutic dosimetry of normal organs and tissues of 177 Lu-PSMA-617 prostate-specific membrane antigen (PSMA) inhibitor in patients with castration-resistant prostate cancer. Eur J Nucl Med Mol Imaging.

[27] Privé BM, Peters SMB, Muselaers CH, van Oort IM, Janssen MJ, Sedelaar M, et al. Lutetium-177-PSMA-617 in low-volume hormone sensitive metastatic prostate cancer, a prospective pilot study. Clin Cancer Res. 2021. 10.1158/1078-0432.CCR-20-4298 33883176

[28] Beauregard J-M, Hofman MS, Kong G, Hicks RJ (2012). The tumour sink effect on the biodistribution of 68 Ga-DOTA-octreotate: implications for peptide receptor radionuclide therapy. Eur J Nucl Med Mol Imaging.

[29] Gaertner FC, Halabi K, Ahmadzadehfar H, Kürpig S, Eppard E, Kotsikopoulos C (2017). Uptake of PSMA-ligands in normal tissues is dependent on tumor load in patients with prostate cancer. Oncotarget.

[30] Afshar-Oromieh A, Malcher A, Eder M, Eisenhut M, Linhart H, Hadaschik B (2013). PET imaging with a [68 Ga] gallium-labelled PSMA ligand for the diagnosis of prostate cancer: biodistribution in humans and first evaluation of tumour lesions. Eur J Nucl Med Mol Imaging.

[31] Dutch Society of Nuclear Medicine. Procedure guidelines nuclear medicine. Part IV: Equipment. Kloosterhof Neer BV; 2016. p. 662–70.

[32] Peters SM, Viol SLM, van der Werf NR, de Jong N, van Velden FH, Meeuwis A (2020). Variability in lutetium-177 SPECT quantification between different state-of-the-art SPECT/CT systems. EJNMMI physics.

[33] Ljungberg M, Celler A, Konijnenberg MW, Eckerman KF, Dewaraja YK, Sjogreen-Gleisner K (2016). MIRD pamphlet no. 26: joint EANM/MIRD guidelines for quantitative 177Lu SPECT applied for dosimetry of radiopharmaceutical therapy. J Nucl Med.

[34] Bolch WE, Eckerman KF, Sgouros G, Thomas SR (2009). MIRD pamphlet no. 21: a generalized schema for radiopharmaceutical dosimetry—standardization of nomenclature. J Nucl Med.

[35] Valentin J (2002). Basic anatomical and physiological data for use in radiological protection: reference values: ICRP Publication 89. Ann ICRP.

[36] Hindorf C, Glatting G, Chiesa C, Lindén O, Flux G (2010). EANM Dosimetry Committee guidelines for bone marrow and whole-body dosimetry. Eur J Nucl Med Mol Imaging.

[37] Buijs WC, Siegel JA, Boerman OC, Corstens FH (1998). Absolute organ activity estimated by five different methods of background correction. J Nucl Med.

[38] Jentzen W (2015). An improved iterative thresholding method to delineate PET volumes using the delineation-averaged signal instead of the enclosed maximum signal. J Nucl Med Technol.

[39] Andersson M, Johansson L, Minarik D, Mattsson S, Leide-Svegborn S (2014). An internal radiation dosimetry computer program, IDAC 2.0, for estimation of patient doses from radiopharmaceuticals. Radiat Prot Dosimetry.

[40] Gear JI, Cox MG, Gustafsson J, Gleisner KS, Murray I, Glatting G (2018). EANM practical guidance on uncertainty analysis for molecular radiotherapy absorbed dose calculations. Eur J Nucl Med Mol Imaging.

[41] Begum NJ, Thieme A, Eberhardt N, Tauber R, D’Alessandria C, Beer AJ (2018). The effect of total tumor volume on the biologically effective dose to tumor and kidneys for 177Lu-labeled PSMA peptides. J Nucl Med..

[42] Deasy JO, Moiseenko V, Marks L, Chao KC, Nam J, Eisbruch A (2010). Radiotherapy dose–volume effects on salivary gland function. Int J Radiat Oncol Biol Phys.

[43] Stewart F, Akleyev A, Hauer-Jensen M, Hendry J, Kleiman N, Macvittie T (2012). ICRP publication 118: ICRP statement on tissue reactions and early and late effects of radiation in normal tissues and organs–threshold doses for tissue reactions in a radiation protection context. Ann ICRP.

[44] Bergsma H, Konijnenberg MW, Van Der Zwan WA, Kam BL, Teunissen JJ, Kooij PP (2016). Nephrotoxicity after PRRT with 177 Lu-DOTA-octreotate. Eur J Nucl Med Mol Imaging.

[45] Wessels BW, Konijnenberg MW, Dale RG, Breitz HB, Cremonesi M, Meredith RF, et al. MIRD pamphlet No. 20: the effect of model assumptions on kidney dosimetry and response—implications for radionuclide therapy. J Nucl Med. 2008;49:1884–99. 10.2967/jnumed.108.053173 18927342

[46] Gundem G, Van Loo P, Kremeyer B, Alexandrov LB, Tubio JMC, Papaemmanuil E (2015). The evolutionary history of lethal metastatic prostate cancer. Nature.

[47] Gerlinger M, Rowan AJ, Horswell S, Larkin J, Endesfelder D, Gronroos E (2012). Intratumor heterogeneity and branched evolution revealed by multiregion sequencing. N Engl J Med.

[48] Greaves M, Maley CC (2012). Clonal evolution in cancer. Nature.

[49] Hänscheid H, Lapa C, Buck AK, Lassmann M, Werner RA (2018). Dose mapping after endoradiotherapy with 177Lu-DOTATATE/DOTATOC by a single measurement after 4 days. J Nucl Med.

[50] Edler von Eyben F, Singh A, Zhang J, Nipsch K, Meyrick D, Lenzo N (2019). (177)Lu-PSMA radioligand therapy of predominant lymph node metastatic prostate cancer. Oncotarget.

[51] Del Prete M, Buteau F-A, Arsenault F, Saighi N, Bouchard L-O, Beaulieu A (2019). Personalized 177 Lu-octreotate peptide receptor radionuclide therapy of neuroendocrine tumours: initial results from the P-PRRT trial. Eur J Nucl Med Mol Imaging.

