# Supplementary material for: Intra-therapeutic dosimetry of [177Lu]Lu-PSMA-617 in low-volume hormone-sensitive metastatic prostate cancer patients and correlation with treatment outcome
Source: Eur J Nucl Med Mol Imaging. 2021 Jul 4;49(2):460–9. doi: 10.1007/s00259-021-05471-4 (PMC8803803; doi:10.1007/s00259-021-05471-4)
Supplement: Supplementary file 1 — Supplementary file1 (DOCX 1215 KB) [file 259_2021_5471_MOESM1_ESM.docx]

# Supplementary materials

**Title: Intra-Therapeutic Dosimetry of Lu-177-PSMA-617 in Low-Volume Hormone Sensitive Metastatic Prostate Cancer Patients and Correlation with Treatment Outcome**

**Journal: European Journal of Nuclear Medicine and Molecular Imaging**

**Authors:** Steffie M.B. Peters^1^, Bastiaan M. Privé^1^, Maarten de Bakker^1^, Frank de Lange^1^, Walter Jentzen^2^, Annemarie Eek^1^, Constantijn H.J. Muselaers^3^, Niven Mehra^4^, J. Alfred Witjes^3^, Martin Gotthardt^1^, James Nagarajah^1^, Mark W. Konijnenberg^1,5^

^1^: Department of Medical Imaging, Radboud university medical center, Nijmegen, The Netherlands

^2^: Department of Nuclear Medicine, University of Duisburg-Essen, Essen, Germany

^3^: Department of Urology, Radboud university medical center, Nijmegen, The Netherlands

^4^: Department of Medical Oncology, Radboud university medical center, Nijmegen, The Netherlands

^5^: Department of Radiology and Nuclear Medicine, Erasmus Medical Center, Rotterdam, The Netherlands

**Corresponding and first author:**

Steffie M.B. Peters (ORCID: 0000-0002-0752-7134)

Radboud university medical center

Department of Medical Imaging

P.O. Box 9101

6500 HB Nijmegen, The Netherlands

T: +31 (6) 11621752

E: steffie.peters@radboudumc.nl


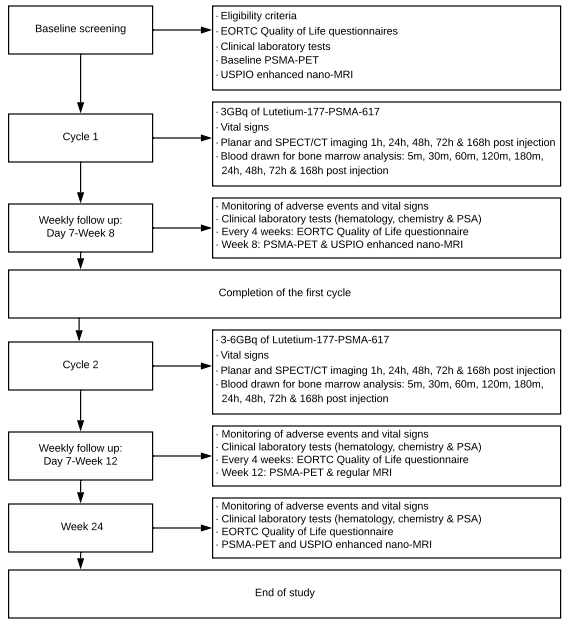


**Figure S1:** Study flowchart

**Materials S1: Uncertainty analysis flowchart**

1. Error in SPECT camera specific calibration factor for ^177^Lu: 5% [Peters, 2020]

CF = 10.6 ± 0.5 cps/MBq

1. Data collection:
   1. Quantitative SPECT at 5 time-points, within 45 minute scan-time
   2. Activity concentration in blood samples at 9 time points; error: 5%
2. Drawing of VOIs in SPECT data over organs and tumor lesions to determine counts:
   1. Large spherical VOI over lesions with background correction; error: 10%
   2. CT based VOI over organs; error 5%
3. Determination of lesion and organ volume:
   1. Lesion volume based on PET/CT and diagnostic CT [Jentzen, 2015];

Voxelisation and resolution error volume with lesion diameter d and voxel size a: $\left[ \frac{\delta V}{V} \right]^{2}=9 \left[ \frac{\delta d}{d} \right]^{2}=1.5\left( \frac{a}{d} \right)^{2}$ [Gear, 2018]

- 1. Organ volumes set at fixed ICRP-89 male phantom values; error 10%

1. Fit to Time-Activity Curve
   1. Mono-exponential fit SPECT TAC when R^2^ > 0.7, determine covariance matrix
   2. Actual SPECT when R^2^ < 0.7 with exponential decay from last time-point
   3. Three-exponential curve fit of blood data, determine covariance matrix
2. Integration over time of the TAC, to determine the time-integrated activity Ã
   1. Mono-exponential integration, error in TIA or Ã:
      $\left[ \frac{\delta\tilde{A}}{\tilde{A}} \right]^{2}=\left[ \frac{\delta A_{0}}{A_{0}} \right]^{2}+\left[ \frac{\delta\lambda_{eff}}{\lambda_{eff}} \right]^{2}-\frac{2 Cov\left( A_{0},\lambda_{eff} \right)}{A_{0}\lambda_{eff}}$
   2. Trapezoid integration method; error:
      $\left[ \frac{\delta\tilde{A}}{\tilde{A}} \right]^{2}=\sum_{i} \left[ \frac{\delta A(t_{i})}{A(t_{i})} \right]^{2}$
   3. Three-exponential integration of blood concentration TAC, error in TIA or Ã:

$$\left[ \frac{\delta\tilde{A}}{\tilde{A}} \right]^{2}=\left[ \frac{\delta A_{0}}{A_{0}} \right]^{2}+{\left[ \frac{\delta P}{P} \right]^{2}+\left[ \frac{\delta\%Fast}{\%Fast} \right]^{2}+\left[ \frac{\delta\lambda_{Fast}}{\lambda_{Fast}+\lambda_{p}} \right]}^{2}+\left[ \frac{\delta\%Slow}{\%Slow} \right]^{2}+\left[ \frac{\delta\lambda_{Slow}}{\lambda_{Slow}+\lambda_{p}} \right]+\left[ \frac{\delta\lambda_{Medium}}{\lambda_{Medium}+\lambda_{p}} \right]-\sum covariances$$

3. Determine volume specific lesion S-factor with power-function on S-values spheres $S=81.7V^{-0.988}$; error:

$$\left[ \frac{\delta S}{S} \right]^{2}=\left( 0.988\frac{\delta V}{V} \right)^{2}$$

4. Absorbed dose calculation with MIRD equation $D=\tilde{A}\times S$; error:

$$\left[ \frac{\delta D}{D} \right]^{2}={\left[ \frac{\delta\tilde{A}}{\tilde{A}} \right]^{2}+\left[ \frac{\delta S}{S} \right]}^{2}$$

**Table S1:** organ absorbed dose ((Gy/GBq) ± error) in organs at risk per patient

| Patient # | Salivary glands | | | Kidneys | | | Liver | | | Bone Marrow | | |
| --- | --- | --- | --- | --- | --- | --- | --- | --- | --- | --- | --- | --- |
|  | Cycle 1 | Cycle 2 | Total | Cycle 1 | Cycle 2 | Total | Cycle 1 | Cycle 2 | Total | Cycle 1 | Cycle 2 | Total |
| 1 | 0.62 ± 0.12 | 0.31 ± 0.04 | 0.41 ± 0.07 | 0.33 ± 0.02 | 0.35 ± 0.04 | 0.34 ± 0.03 | 0.08 ± 0.004 | 0.07 ± 0.002 | 0.08 ± 0.003 | 0.012 ± 0.001 | 0.015 ± 0.006 | 0.014 ± 0.001 |
| 2 | 0.38 ± 0.19 | 0.16 ± 0.01 | 0.23 ± 0.07 | 0.78 ± 0.07 | 0.60 ± 0.05 | 0.66 ± 0.06 | 0.14 ± 0.003 | 0.07 ± 0.003 | 0.09 ± 0.003 | 0.020 ± 0.02 | 0.022 ± 0.007 | 0.021 ± 0.002 |
| 3 | 0.60 ± 0.10 | 0.28 ± 0.04 | 0.60 ± 0.08 | 0.59 ± 0.02 | 0.47 ± 0.03 | 0.61 ± 0.03 | 0.12 ± 0.003 | 0.10 ± 0.002 | 0.11 ± 0.003 | 0.014 ± 0.004 | 0.015 ± 0.004 | 0.014 ± 0.004 |
| 4 | 0.54 ± 0.08 | 0.47 ± 0.09 | 0.49 ± 0.08 | 0.39 ± 0.03 | 0.35 ± 0.02 | 0.36 ± 0.02 | 0.07 0.006 | 0.07 ± 0.004 | 0.07 ± 0.005 | 0.012 ± 0.005 | 0.014 ± 0.020 | 0.013 ± 0.005 |
| 5 | 0.43 ± 0.06 | 0.30 ± 0.04 | 0.34 ± 0.05 | 0.56 ± 0.03 | 0.68 ± 0.19 | 0.64 ± 0.14 | 0.12 ± 0.003 | 0.12 ± 0.01 | 0.12 ± 0.009 | 0.020 ± 0.002 | 0.020 ± 0.002 | 0.023 ± 0.002 |
| 6 | 0.84 ± 0.11 | 0.57 ± 0.09 | 0.66 ± 0.10 | 0.47 ± 0.02 | 0.30 ± 0.01 | 0.36 ± 0.01 | 0.10 ± 0.002 | 0.09 ± 0.002 | 0.09 ± 0.002 | 0.016 ± 0.002 | 0.019 ± 0.002 | 0.018 ± 0.002 |
| 7 | 0.33 ± 0.03 | 0.14 ± 0.05 | 0.21 ± 0.05 | 0.73 ± 0.06 | 0.35 ± 0.01 | 0.50 ± 0.03 | 0.09 ± 0.003 | 0.08 ± 0.002 | 0.09 ± 0.003 | 0.018 ± 0.008 | 0.018 ± 0.003 | 0.018 ± 0.010 |
| 8 | 0.31 ± 0.02 | 0.51 ± 0.05 | 0.44 ± 0.04 | 0.59 ± 0.03 | 0.40 ± 0.04 | 0.46 ± 0.03 | 0.10 ± 0.003 | 0.07 ± 0.007 | 0.08 ± 0.006 | 0.019 ± 0.007 | 0.021 ± 0.004 | 0.020 ± 0.006 |
| 9 | 0.31 ± 0.09 | 0.34 ± 0.08 | 0.33 ± 0.08 | 0.46 ± 0.04 | 0.57 ± 0.03 | 0.53 ± 0.03 | 0.10 ± 0.004 | 0.10 ± 0.004 | 0.10 ± 0.005 | 0.015 ± 0.011 | 0.014 ± 0.003 | 0.014 ± 0.010 |
| 10 | 0.19 ± 0.02 | 0.11 ± 0.02 | 0.14 ± 0.02 | 0.78 ± 0.06 | 0.33 ± 0.02 | 0.48 ± 0.03 | 0.12 ± 0.002 | 0.08 ± 0.002 | 0.09 ± 0.002 | 0.016 ± 0.007 | 0.018 ± 0.012 | 0.017 ± 0.010 |
| Mean + SD | 0.46 ± 0.19 | 0.32 ± 0.16 | 0.39 ± 0.17 | 0.57 ± 0.16 | 0.44 ± 0.13 | 0.49 ± 0.11 | 0.10 ± 0.02 | 0.08 ± 0.02 | 0.09 ± 0.01 | 0.016 ± 0.003 | 0.016 ± 0.006 | 0.017 ± 0.008 |
| Median | 0.41 | 0.30 | 0.38 | 0.57 | 0.37 | 0.49 | 0.10 | 0.08 | 0.09 | 0.016 | 0.016 | 0.018 |
| Range | 0.19 – 0.84 | 0.11 – 0.57 | 0.14 – 0.66 | 0.33 – 0.78 | 0.30 – 0.68 | 0.34 – 0.66 | 0.07 – 0.14 | 0.07 ± 0.12 | 0.07 – 0.12 | 0.012 – 0.020 | 0.009 – 0.022 | 0.013 – 0.023 |

SD: standard deviation

**Figure S2:** Blood measurements for organ toxicity. Dotted vertical lines indicate the first and second therapeutic cycle with ^177^Lu-PSMA-617. (a-c): hemoglobin, white blood cells and thrombocytes, indicators for blood and bone marrow toxicity; (d): creatinine, indicator for kidney toxicity; (e-f): ALAT and ASAT, indicators for liver toxicity.

**Table S2:** Overview of SUV_max_, tumor volumes and absorbed dose in lesions ((Gy/GBq) or (Gy) ± error). Values in bold are for target lesions.

|  |  | Cycle 1 | | | Cycle 2 | | |  |
| --- | --- | --- | --- | --- | --- | --- | --- | --- |
| Patient # | Type of lesion | SUV_max_ PET | Tumor volume (ml) | Absorbed dose (Gy/GBq) | SUV_max_ PET | Tumor volume (ml) | Absorbed dose (Gy/GBq) | Total (Gy) |
| 1 | **LN** | **11.5** | **0.27** | **2.21 ± 1.49** | **4.0** | **Not visible** | **not visible** | **6.66 ± 4.50** |
| 2 | B | 9.3 | 0.50 | 1.97 ± 0.61 | 16.2 | 1.22 | 0.58 ± 0.23 | 9.79 ± 3.29 |
|  | **B** | **31.3** | **3.48** | **3.67 ± 0.74** | **43.7** | **2.99** | **2.75 ± 0.50** | **28.23 ± 5.35** |
|  | B | 5.4 | 0.61 | 0.41 ± 0.13 | 11.5 | 0.56 | 0.24 ± 0.07 | 2.75 ± 0.87 |
| 3 | **LN** | **44.7** | **1.45** | **3.20 ± 0.58** | **34.5** | **1.21** | **3.02 ± 2.04** | **28.08 ± 14.10** |
| 4 | LN | 12.2 | 2.80 | 1.27 ± 0.50 | 17.5 | 3.03 | 1.12 ± 0.27 | 10.67 ± 3.13 |
|  | **B** | **36.3** | **7.52** | **0.95 ± 0.12** | **26.3** | **10.61** | **0.65 ± 0.38** | **6.84 ± 2.64** |
|  | B | 14.8 | 0.43 | 3.00 ± 0.58 | 27.0 | 0.43 | 2.38 ± 1.21 | 23.55 ± 9.09 |
|  | B | 20.8 | 42.49 | 1.90 ± 0.23 | 23.7 | 41.72 | 1.86 ± 0.23 | 17.09 ± 2.08 |
|  | B | 17.1 | 2.98 | 1.05 ± 0.34 | 18.5 | 1.52 | 0.83 ± 0.23 | 8.23 ± 2.45 |
|  | B | 21.5 | 20.21 | 0.93 ± 0.15 | 18 | 20.34 | 1.20 ± 0.11 | 10.11 ± 1.13 |
|  | B | 12.3 | 0.13 | 0.75 ± 0.28 | 7.6 | 0.34 | 2.05 ± 0.78 | 14.66 ± 5.57 |
| 5 | LN | 12.5 | 1.85 | 1.01 ± 0.26 | 17.3 | 0.66 | 7.93 ± 1.20 | 50.46 ± 7.97 |
|  | LN | 8.1 | 0.69 | 0.70 ± 0.12 | 8.3 | 0.43 | 2.08 ± 1.08 | 14.52 ± 6.78 |
|  | **LN** | **22.8** | **1.07** | **2.39 ± 0.37** | **18.0** | **0.72** | **6.51 ± 0.84** | **46.14 ± 6.15** |
|  | LN | 11.8 | 0.44 | 0.71 ± 0.10 | 11.0 | 0.51 | 3.41 ± 0.74 | 22.54 ± 4.76 |
|  | LN | 9.6 | 0.32 | 0.52± 0.21 | 6.5 | 0.58 | 1.52 ± 0.25 | 10.68 ± 2.09 |
| 6 | **LN** | **11.8** | **1.05** | **4.29 ± 1.10** | **20.8** | **0.49** | **7.75 ± 3.55** | **59.31 ± 24.51** |
|  | LN | 4.5 | 0.68 | 1.48 ± 0.24 | 6.5 | 0.13 | 14.95 ± 7.59 | 93.78 ± 46.07 |
| 7 | **LN** | **11.7** | **0.19** | **4.03 ± 1.13** | **5.9** | **0.30** | **0.47 ± 0.18** | **14.65 ± 4.33** |
|  | LN | 6.5 | 2.68 | 0.57 ± 0.15 | 5.6 | 0.06 | not visible | 1.74 ± 0.45 |
| 8 | **LN** | **33.4** | **1.61** | **4.30 ± 0.45** | **24.2** | **0.39** | **8.06 ± 2.52** | **61.97 ± 16.62** |
| 9 | **LN** | **44.4** | **0.70** | **10.34 ± 2.74** | **33.4** | **0.33** | **15.35 ± 5.15** | **123.48 ± 39.19** |
|  | LN | 7.4 | 0.21 | 3.04 ± 1.23 | 5.5 | 0.005 | not visible | 9.24 ± 3.75 |
| 10 | **B** | **22.3** | **2.00** | **2.04 ± 0.26** | **29.0** | **3.76** | **1.02 ± 0.11** | **12.37 ± 1.45** |
|  | LN | 27.2 | 0.55 | 1.43 ± 0.51 | 21.5 | 0.35 | 6.08 ± 2.12 | 41.08 ± 14.33 |
| Mean + SD |  |  | 3.73 ± 8.86 | 2.22 ± 2.06 |  | 3.71 ± 9.05 | 3.99 ± 4.43 | 28.02 ± 29.50 |
| Median |  |  | 0.88 | 1.69 |  | 0.56 | 2.08 | 14.66 |
| Range |  |  | 0.13 – 42.49 | 0.41 – 10.34 |  | 0.05 – 41.72 | 0.24 – 15.35 | 1.74 – 123.48 |

LN: lymph node. B: Bone.


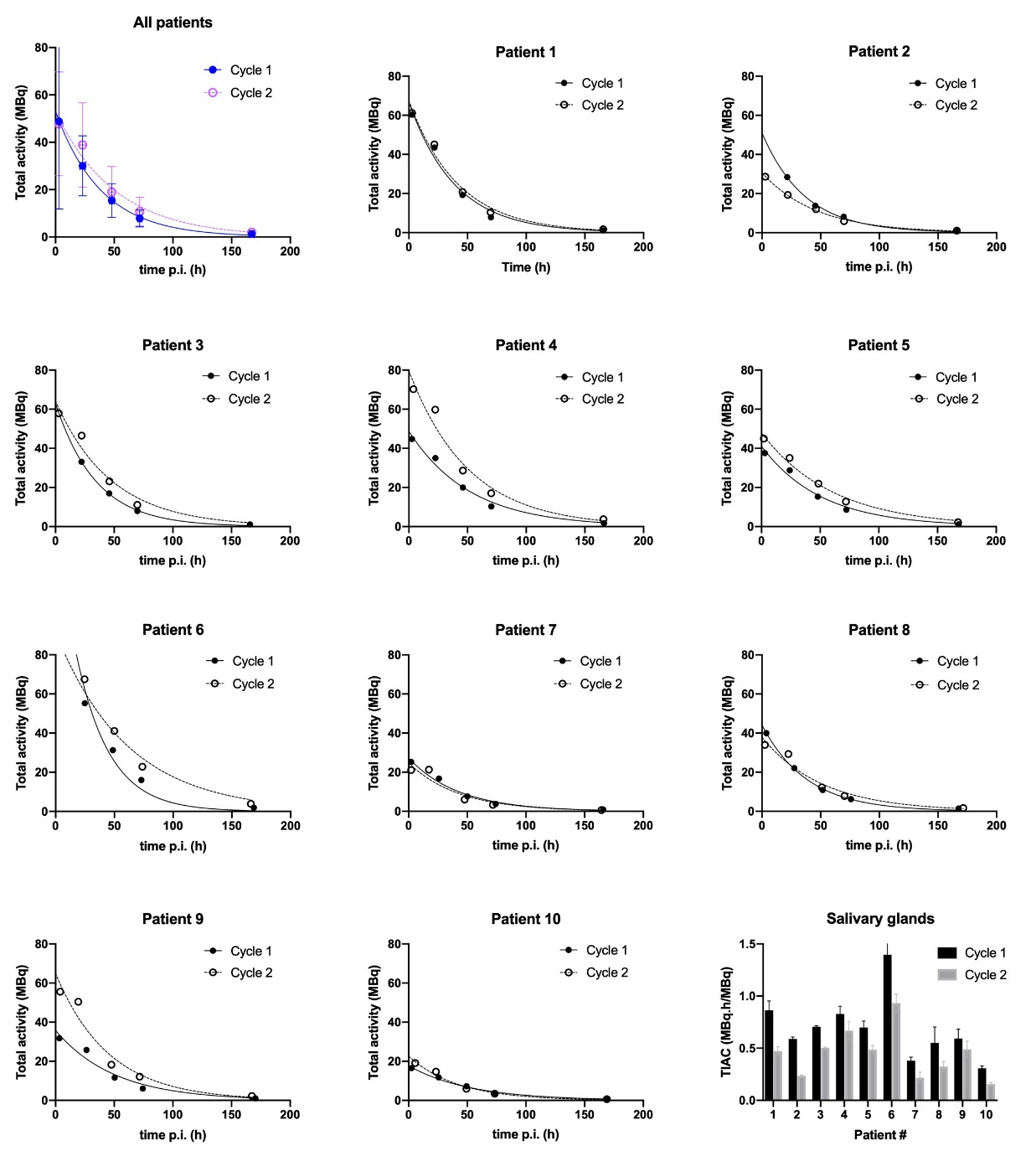


**Figure S3:** ^177^Lu-PSMA-617 kinetics in the salivary glands. The time integrated activity curve (MBq·h/MBq) was lower after the second treatment for all patients.


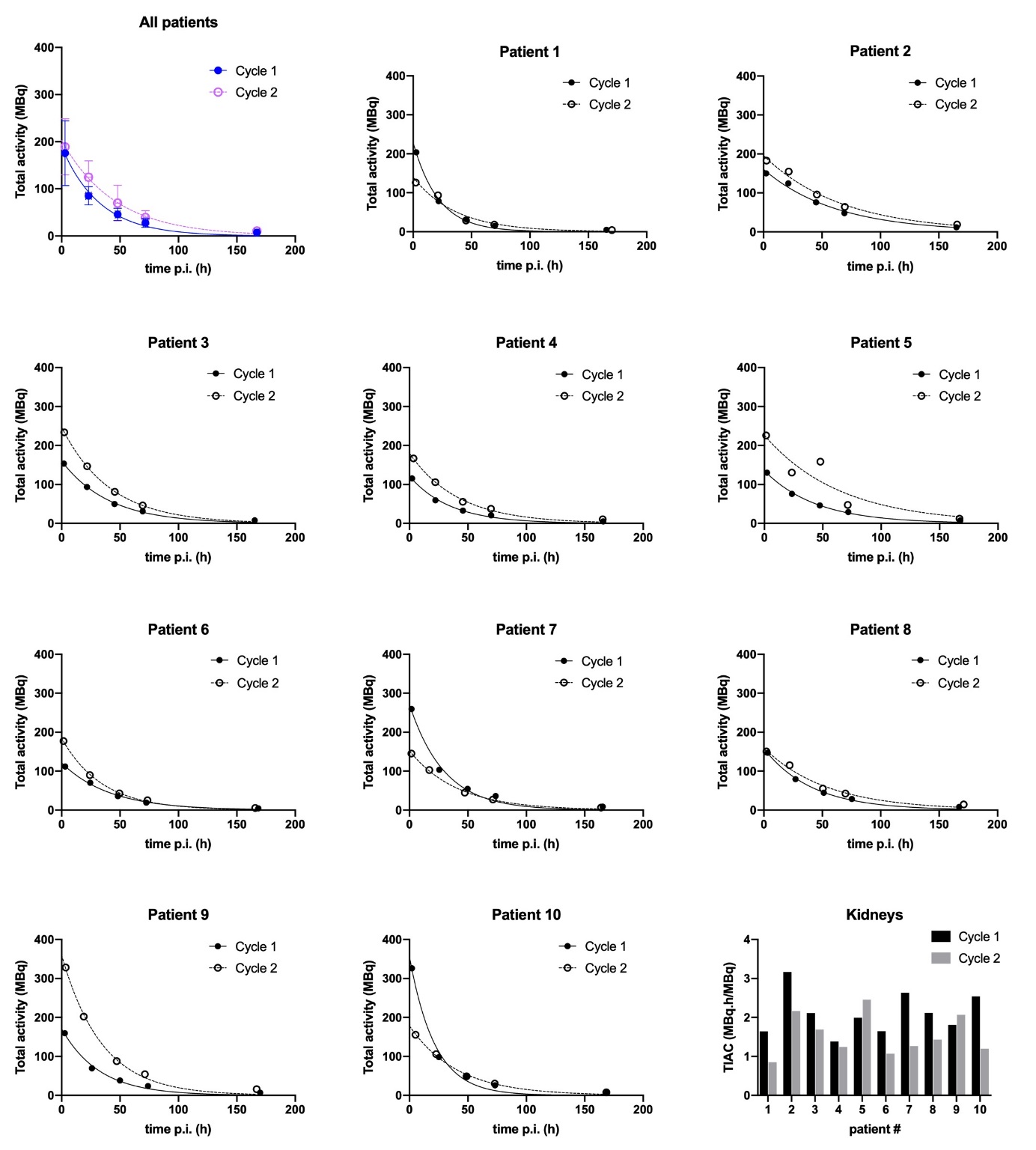


**Figure S4:** ^177^Lu-PSMA-617 kinetics in the kidneys. The time integrated activity curve (MBq·h/MBq) was lower after the second treatment for most patients.


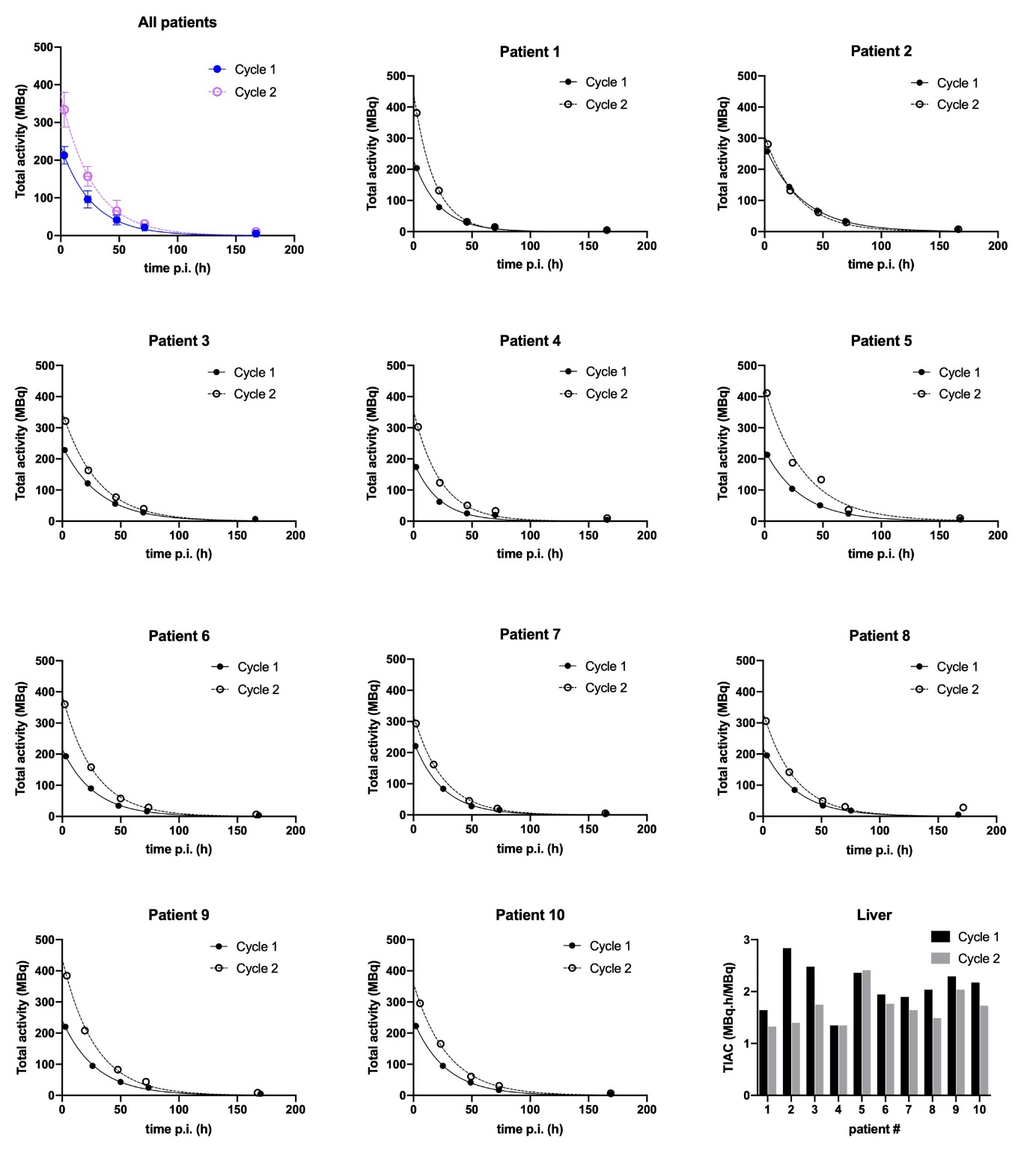


**Figure S5:** ^177^Lu-PSMA-617 kinetics in the liver. The time integrated activity curve (MBq·h/MBq) was lower after the second treatment for most patients.

| Organ | Threshold Dose (Gy) | Absorbed dose found in this study (Gy/GBq) | Activity at which threshold dose will be reached (GBq) |
| --- | --- | --- | --- |
| Salivary glands | 25^1^ | Min: 0.14 | 178 |
|  |  | Max 0.66 | 38 |
| Kidneys | 40^2^ | Min: 0.34 | 118 |
|  |  | Max: 0.66 | 61 |
| Liver | 30^3^ | Min: 0.07 | 429 |
|  |  | Max: 0.12 | 250 |
| Bone Marrow | 2^3^ | Min: 0.013 | 154 |
|  |  | Max: 0.023 | 87 |

**Table S3:** comparison between the threshold dose for each organ and the absorbed dose found in this study. Based on these two parameters it is possible to make a prediction on a safe activity to administer without risking healthy organ damage.

^1^: Deasy et al. 2010 [42]; ^2^: Bergsma et al. 2016 [44] and Wessels et al. 2008 [45]; ^3^: Stewart et al. 2012 [43].

**Materials S2: Evaluation of dose estimation of treatment cycle 2.**

An evaluation was performed to compare the absorbed dose in cycle 2 determined based on the complete 5 time point data set, with an estimation based on a single data point. Using the cumulated activity of one time point in cycle 2 and the effective half-life of [^177^Lu]Lu-PSMA of the first treatment cycle, the absorbed dose was estimated for the salivary glands and index lesions.


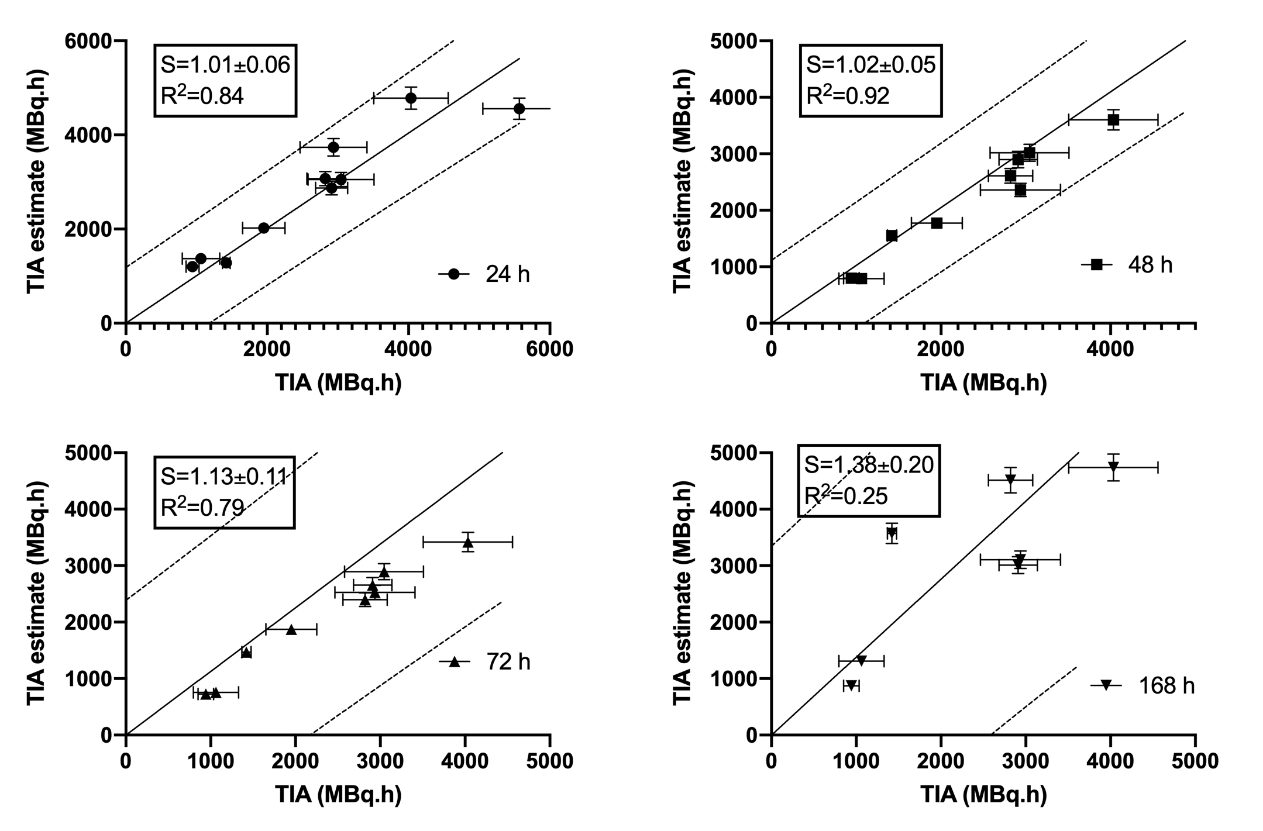


**Figure A:** Comparison between the time integrated activity based on the complete dataset of cycle 2 and the estimated time integrated activity based on a single time point for the salivary glands. The 24 and 48 hour time points could serve as an reliable estimation of the actual time integrated activity and could therefore help to decrease the necessary data points. S = slope, R^2^ = goodness of fit.


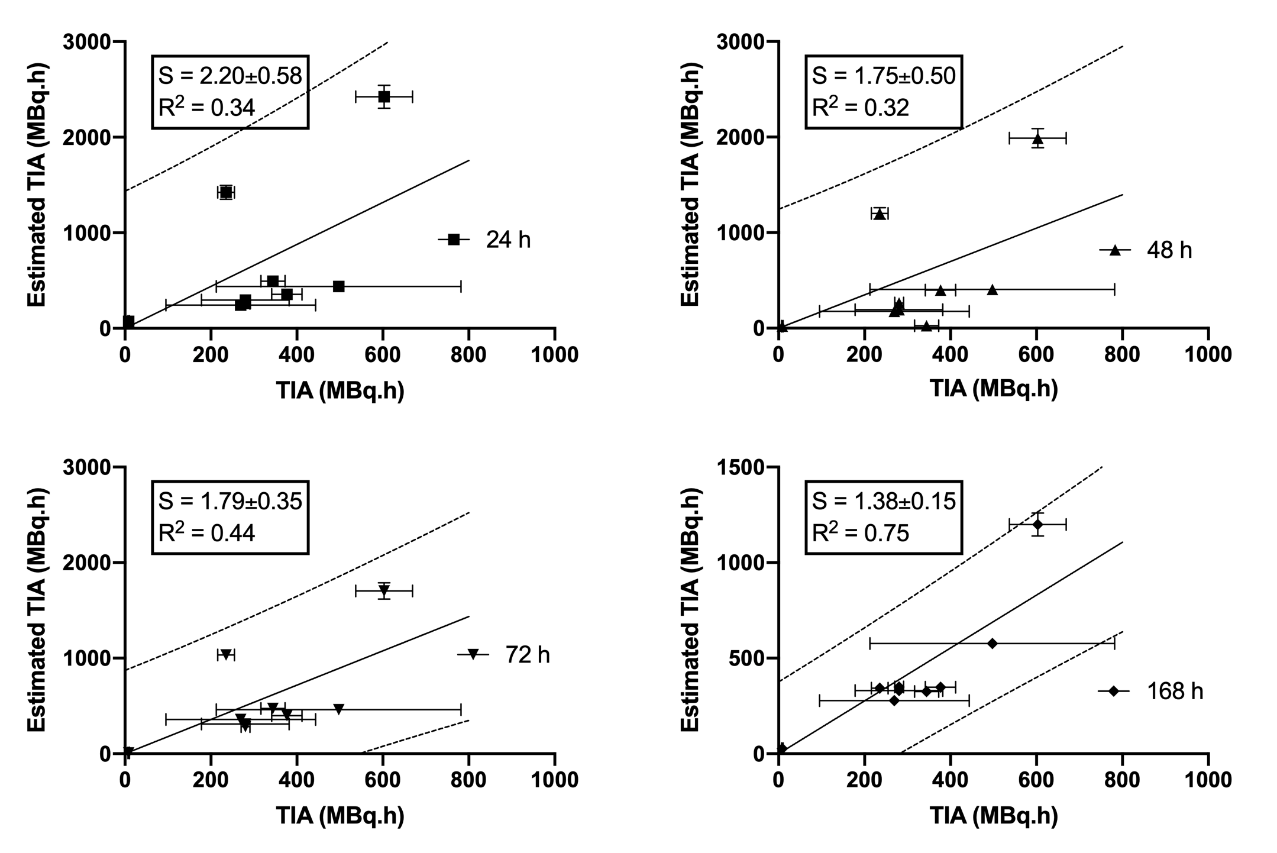


**Figure B:** Comparison between the time integrated activity based on the complete dataset of cycle 2 and the estimated time integrated activity based on a single time point for the index lesions. As can be seen the lesion kinetics vary considerably, therefore a reliable fit is difficult to obtain. For lesions it might be necessary to have data of more than one time point. S = slope, R^2^ = goodness of fit.
